# Sense of body ownership and body agency in schizophrenia

**DOI:** 10.1038/s41598-022-23988-y

**Published:** 2022-11-12

**Authors:** Ileana Rossetti, Martina Repossi, Vincenzo Florio, Benedetta Demartini, Andreas Conca, Orsola Gambini, Angelo Maravita

**Affiliations:** 1 Dipartimento di Psicologia, Università di Milano-Bicocca, Piazza dell’Ateneo Nuovo 1, 20126 Milan, Italy; 2 Dipartimento di Psichiatria, Comprensorio Sanitario di Bolzano, Via Lorenz Böhler 5, 39100 Bolzano, Italy; 3 Dipartimento di Scienze della Salute, Università degli Studi di Milano, Milan, Italy; 4 Unità Di Psichiatria II, A.O. San Paolo, ASST Santi Paolo e Carlo, Milan, Italy; 5 Aldo Ravelli” Research Center for Neurotechnology and Experimental Brain Therapeutics, Università degli Studi di Milano, Milan, Italy; 6 Neuromi—Milan Center for Neuroscience, University of Milano-Bicocca, Milan, Italy

**Keywords:** Human behaviour, Consciousness, Perception

## Abstract

Recent research suggests that embodiment sensations (sense of body ownership and sense of body agency) are altered in schizophrenia. Using a mirror box illusion setup, we tested if the anomalous embodiment experience depends on deficient processing of visuomotor synchrony, disrupted processing of movement mode, or both. The task required participants to press a lever with their index while looking at the image of the experimenter’s hand moving on a similar lever. The illusion of embodiment could arise because looking toward the direction of their own hand the participant saw the reflection of the experimenter’s hand visually superimposed to his own one through a mirror. During the illusion induction, we systematically varied visuomotor asynchrony (4 delays were imposed on the movement of the experimenter’s hand) and the mode of movement (the participant could perform active vs. passive movements). The strength of the illusion of embodiment of the external hand was assessed with explicit judgments of ownership and agency. Patients’ data showed an anomalous modulation of ownership with respect to visuomotor synchrony manipulation and an altered modulation of agency with respect to both visuomotor synchrony and movement mode manipulations. Results from the present study suggest that impairments affecting both the processing of temporal aspects of visuomotor signals and the processing of type of movement underlie anomalous embodiment sensations in schizophrenia. Hypotheses about potential deficits accounting for our results are proposed.

## Introduction

Sense of body Ownership (SoO) and Sense of body Agency (SoA) are two core aspects of body awareness. SoO is in the experience of mine-ness we have towards a body part, while SoA is the experience of motor control over body actions. SoO and SoA are rooted in the integration of sensory information regarding the body from different sensory channels^1^. Much empirical evidence comes from embodiment illusion experiments capitalizing on visuo-tactile stimulation. The most classic example is the Rubber Hand Illusion, RHI (but see also more recent virtual reality paradigms, e.g.^2^), where simultaneous touches on a mannequin’s hand and participant’s hand^3^ can elicit the illusion of SoO over the fake hand. However, illusions of embodiment can be induced through a variety of multimodal stimulation^4,5^, including visuomotor stimulation, e.g., the moving RHI^6–8^, wherein the illusion of embodiment is elicited during the execution of hand movements.

As forms of first-person self-reference^9^ present in any daily activity on the pre-reflective level, SoO and SoA play a major role in the experience of Self^10^. Based on the link between schizophrenia and self-disturbances, SoO and SoA have been explored in patients diagnosed with this psychiatric disorder over the past decades^11^. A number of RHI studies shows abnormal SoO in schizophrenia^12–16^ (but see^17^), suggesting unbalanced multisensory integration and/or weaker pre-existing body templates^18,19^ along with a shallower peripersonal space representation^20^. In addition, aberrancies of SoO were found to correlate with the severity of hallucinations and delusions^12–14^, passivity symptoms^15^ and anhedonia^16^. Additional evidence about SoO and SoA in schizophrenia comes from visuomotor body illusions experiments^21–23^. In a previous study based on the Mirror Box Illusion (MBI), patients’ SoA did not significantly modulated according to the degree of visuomotor congruence experimentally manipulated^23^. Other studies focused on the relation between aberrant SoO/SoA and passivity (also Schneiderian first-rank) symptoms, a subset of positive symptoms particularly related to “the sense of being passive”^24^. Patients suffering from these symptoms may experience their own thoughts, actions, feelings or body sensations as being made, influenced or manipulated by external forces or agents (e.g. delusions of control). These psychopathological manifestations suggested that mechanisms for self-ascription of body experiences may be more severely impaired in these patients. For instance, vulnerability to passivity has been associated with impaired proprioceptive cues to SoA over one’s own hand^21^ and with a bidirectional SoO-SoA deficit such that the lack of SoA induces a greater lack of SoO, and vice versa^22^.

In the current study, we sought to deepen the role of two processes potentially affecting the attribution of body sensations in schizophrenia, that is the detection of asynchrony between visuomotor signals and the processing of movement mode (active vs. passive). Previous moving RHI studies with healthy individuals demonstrated that both these factors account for the modulation of SoO and SoA over an external hand. Specifically, the degree of visuomotor synchrony significantly influences SoO while the execution of active or passive movements is less crucial. On the other hand, both factors impact on SoA. SoA is gradually reduced by increasing visuomotor asynchrony and it is almost abolished by passive movements^6,8^. To address these aspects in patients with schizophrenia, we used MBI setup to elicit the illusion of embodiment of an external hand during active and passive movements and with different degrees of temporal incongruence (4 visuomotor delays).

Based on findings suggesting higher visuomotor mismatch detection threshold in schizophrenia^25^, we expected that the experimental manipulation of visuomotor synchrony had a smaller influence on patients’ SoO and SoA as compared to healthy participants. Moreover, assuming a more dramatic derangement of self-attribution processes in patients suffering from passivity experiences, we hypothesized a significant moderation effect of passivity symptoms severity on SoA judgments in the schizophrenia group. In other terms, we expected that patients suffering from more severe passivity symptoms showed more similar SoA ratings between active and passive conditions than patients with a less severe passivity profile. The effect of antipsychotic medication was also analysed in an exploratory way.

## Results

Significant interaction effects resulting from F-tests of the best fitting models are reported hereafter. For the complete output of analyses, see Supplementary Tables 1, 2 and 3. Interaction effects are interpreted by adjusted means (± 95% confidence interval) and graph inspection.

### SoO

**Figure 1:**
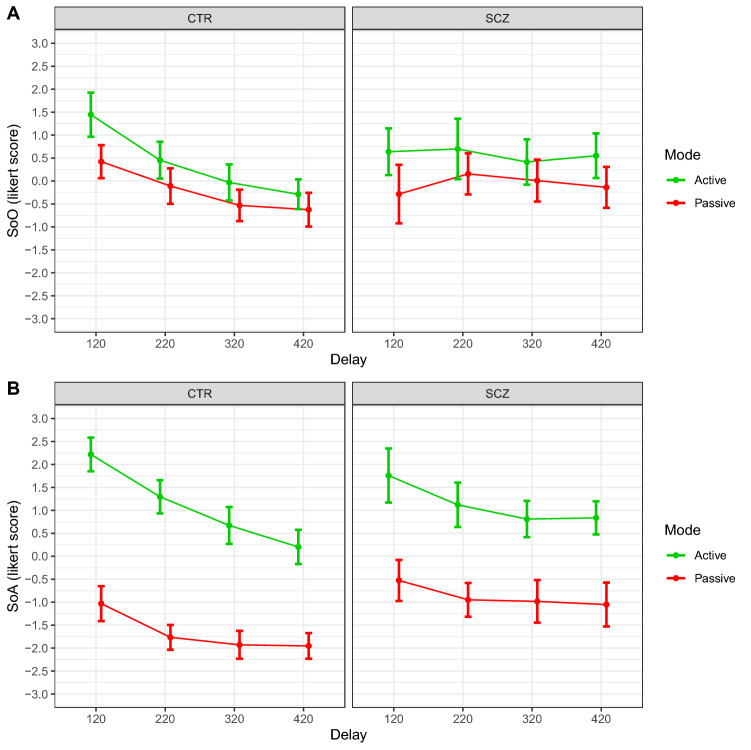
SoO and SoA scores. The graph illustrates mean ratings by group and experimental condition ((**A**) SoO, (**B**) SoA). Green and red lines represent active and passive conditions respectively. Error bars = 95% confidence intervals.

The significant interaction effect *Delay* × *Group* (${F}_{\mathrm{3,420}}=8.091, p<.001$) suggested that the modulation of SoO across different delays was different between groups. Figure 1, Panel A shows that SoO gradually decreases while visuomotor asynchrony increases in controls. Conversely, SoO does not noticeably decline in the patient group. [HC_120_ = 0.934 ± 0.536; HC_220_ = 0.172 ± 0.536; HC_320_ = −0.281 ± 0.536; HC_420_ = −0.457 ± 0.536; SZ_120_ = 0.177 ± 0.605; SZ_220_ = 0.427 ± 0.605; SZ_320_ = 0.211 ± 0.605; SZ_420_ = 0.207 ± 0.605].

### SoO-control

The best fitting model for SoO-control scores included no fixed-effect but *Mode*. This effect did not reach significance (${F}_{\mathrm{1,426}}=2.897, p= .09$). Therefore, unlikely SoO sensations (e.g., having more than one hand), if present, were not influenced by the experimental manipulations. Since SoO-control scores were towards negative values in all conditions and in both groups (see Supplementary Information, section S1.3), we excluded that participants reported SoO sensations because of compliancy or suggestibility.

### SoA

The interaction effect *Mode* × *Delay* (${F}_{\mathrm{3,416}}=3.961, p<.01$) was significant, indicating that SoA modulation dependent on delays was different between movement modes. This effect suggested that SoA declined at larger delays in the active conditions, but differences driven by visuomotor asynchrony were smoothed out in case of passive movements [Active_120_ = 2.011 ± 0.378; Active_220_ = 1.226 ± 0.378; Active_320_ = 0.74 ± 0.378; Active_420_ = 0.477 ± 0.378; Passive_120_ = −0.802 ± 0.378; Passive_220_ = −1.39 ± 0.378; Passive_320_ = −1.482 ± 0.378; Passive_420_ = −1.498 ± 0.378].

The significant and marginally significant effects *Mode* × *Group* (${F}_{\mathrm{1,416}}=14.807, p<.001$) and *Delay* × *Group* (${F}_{\mathrm{3,416}}=2.598, p=.052$) indicated that SoA modulations due to the two factors (especially *Mode of Movement*) were different in the patient group. As can be seen in Fig. 1, Panel B, SoA during active conditions is significantly higher as compared to passive conditions in the control group, however this difference is considerably smaller in the clinical group. Moreover, SoA gradually decreases at larger delays in the control group, however this decline is less marked in patients [HC_active_ = 1.098 ± 0.404; HC_passive_ = −1.67 ± 0.404; SZ_active_ = 1.131 ± 0.444; SZ_passive_ = −0.877 ± 0.444 – HC_120_ = 0.594 ± 0.437; HC_220_ = −0.234 ± 0.437; HC_320_ = −0.629 ± 0.437; HC_420_ = −0.875 ± 0.437; SZ_120_ = 0.616 ± 0.495; SZ_220_ = 0.086 ± 0.495; SZ_320_ = −0.086 ± . 495; SZ_420_ = −0.108 ± 0.495].

### SoA-control

**Figure 2:**
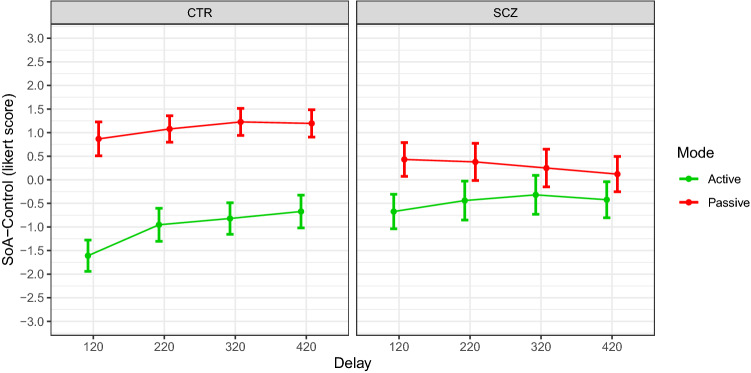
SoA-Control scores. The graph illustrates mean questionnaire ratings by group and experimental condition. Green and red lines represent active and passive conditions respectively. Error bars = 95% confidence intervals.

The significant effect *Mode* × *Group* (${F}_{\mathrm{1,416}}=59.707, p<.001$) and the marginally significant effect *Delay* × *Group* (${F}_{\mathrm{3,416}}=2.604, p=.052$) indicated that the delay and the mode of movement impacted on SoA-control scores differently between groups. Figure 2 shows that SoA-control scores are significantly higher in passive than active conditions in controls. This difference is noticeably smaller in patients. Moreover, scores tend to increase at larger delays in the control group, but not in the patient group [HC_active_ = −1.014 ± 0.344; HC_passive_ = 1.092 ± 0.344; SZ_active_ = −0.463 ± 0.372; SZ_passive_ = 0.295 ± 0.372—HC_120_ = −0.371 ± 0.377; HC_220_ = 0.063 ± 0.377; HC_320_ = 0.203 ± 0.377; HC_420_ = 0.262 ± 0.377; SZ_120_ = −0.121 ± 0.416; SZ_220_ = −0.03 ± 0.416; SZ_320_ = −0.034 ± 0.416; SZ_420_ = −0.151 ± 0.416].

Exploratory correlations show significant associations between SoO and SoA in both groups and in all experimental conditions (Supplementary Figs. 1 and 2).

### Psychopathology and antipsychotic treatment

Passivity index, included as fixed covariate in the model of every questionnaire component, survived model reduction procedure only with SoO-control and SoA-control data. The F-test on SoO-control scores did not show any significant effect (Supplementary Table 2). The F-test on SoA-control scores showed instead the significant effects of *Mode* (${F}_{\mathrm{1,201}}=33.497, p<.001$) and *Mode* × *Passivity* (${F}_{\mathrm{1,201}}=6.867, p<.01$). This interaction effect indicated that patients with more severe passivity profile tended to rate less positively SoA-control items in passive conditions than patients with less severe passivity profile. However, normality test on conditional residuals of the model was significant ($W=.987, p=.03$).

The olanzapine equivalent dose, included as fixed covariate in the model of every questionnaire component, survived model reduction procedure with SoO and SoA data. As for SoO, the main effect *Olanzapine* was marginally significant (${F}_{\mathrm{1,27}}=4.659, p=.04$) suggesting that patients assuming higher doses of neuroleptic agents provided generally higher SoO scores (Supplementary Fig. 3). No other significant effect related to pharmacological treatment was found.

In patients, correlation matrices did not reveal any relevant association between SoO/SoA and psychopathological subscales except for a significant correlation between SoA in the active condition with 320 ms delay and affective flattening (Supplementary Fig. 2).

## Discussion

The aim of the present study was to explore whether mechanisms underlying SoO and SoA are impaired in schizophrenia, with a particular focus on the processing of visuomotor synchrony and movement mode. In this regard, our study yielded two main findings. First, a defective processing of visuomotor asynchrony affects the modulation of patients’ SoO and SoA judgments. Second, an altered processing of movement mode impacts on the modulation of SoA, but not on the modulation of SoO judgments.

To make judgments of SoA and SoO we can rely on different indicators of “mineness”. In other words, we may draw on non-conceptual cues and/or on cognitive cues to provide judgments of SoO and SoA^26^. On the one hand, SoA judgments can be based on perceptual and sensorimotor information about actions (e.g., the outcome of the comparison between motor predictions and actual sensory feedback when a self-generated action is performed; premotor signals). On the other hand, SoA judgments may also depend on cognitive cues, like impersonal background beliefs about authorship, e.g., “the fact of being the only possible cause in the region of interest makes me the cause”^26^. Correspondingly, SoO judgments can be based on perceptual and top-down information about our body (e.g., the outcome of the comparison between proprioception and visual feedback; the result of the comparison between somatic signals and pre-existing internal representation of the body)^26^. However, SoO judgments may also depend on cognitive cues, like impersonal beliefs about SoO, e.g., “Nobody else but me can own my hand”. The processing of both types of cue may be impaired in patients because both anomalous sensory experiences and aberrant belief formation can be assumed in schizophrenia^24^. For this reason, both perceptual and cognitive deficits will be taken into account to motivate the anomalous modulation of SoO and SoA judgments of patients in our study.

Our results revealed that SoO and SoA judgments are significantly reduced by the perturbation of visuomotor synchrony, as can be observed in healthy participants’ data. This finding corroborates previous empirical evidence suggesting that temporal congruence between multimodal signals is important for embodiment sensations^4^. As regards visuomotor illusion, it was found that SoO and SoA were significantly higher when the delay imposed on the visual feedback was smaller than 200 ms^27^. As expected, the manipulation of visuomotor synchrony had a smaller impact on SoO and SoA judgments in the patient group. Differently from controls, patients’ SoO did not remarkably decrease from positive to negative values when larger delays were added to the visual feedback (that is, the movement of the hand in the mirror). Similarly, patients’ SoA declines less steeply than controls’ SoA. These results are coherent with the suggestion from a previous study that schizophrenia patients have a higher visuomotor mismatch detection threshold^25^. In that study, participants were asked to judge whether the image of a virtual hand (visually superimposed to their own real one) precisely replicated their own movements, while small to large delays were introduced. Patients did not clearly detect the temporal distortion of the visual feedback (i.e., the image of the virtual hand) up to 300 ms delay compared to 150 ms delay in healthy controls^25^. Current results partially replicate those from a previous MBI study in schizophrenia. In that study, patients’ SoA was less strongly modulated by the degree of visuomotor asynchrony as compared to healthy controls. However, patients’ SoO did not significantly differ from controls’ SoO^23^. We suggest that different SoO results in the present study may be due to differences in the experimental design, such as the introduction of four delay conditions ranging from 120 to 420 ms at 100-ms intervals that may have determined the anomalous modulation of SoO to more explicitly manifest.

The reduced capability to detect visuomotor temporal conflicts may depend on several low-level non-conceptual processes that may be dysfunctional in schizophrenia. On the one hand, the reduced modulation of SoO and SoA by experimental delays may be due to an widened temporal binding window (for a review^28^). This impairment would make patients unable to detect temporal incongruencies between visuomotor signals at larger delays. Accordingly, they may perceive synchrony when healthy controls do not^21^. On the other hand, the reduced modulation of SoO and SoA by experimental delays may be caused by a critical unreliability of internal signals, like proprioceptive reafference and motor predictions^29^. In this regard, a general sensorimotor predictive aberrancy has been suggested, based on the evidence of less sensitivity to both temporal and spatial sensorimotor conflicts in schizophrenia patients^2,30^. Because of the unreliability of internal signals (e.g., proprioception), the perceptual system would be more strongly reliant on exteroceptive feedback (e.g., visual feedback)^29^ and for this reason patients would be insensitive to visuomotor asynchrony.

To sum up, the modulation of patients’ SoO and SoA is less strongly driven by the degree of visuomotor synchrony in the present study. These results suggest that visuomotor synchrony represents a less strong cue to SoO and SoA in schizophrenia. Current findings may be accounted by impairments in low-level mechanisms serving the processing of temporal aspects of visuomotor information (but consider also other potential cognitive impairments discussed below).

The current study also shows that SoA is more intense during active movements as compared to passive ones. This result was expected in healthy controls because the movement is clearly not self-generated during passive conditions. Present finding is also in line with previous moving RHI studies indicating that passive movement can almost extinguish SoA in healthy subjects^6,8^. The comparison between active and passive movement conditions was aimed at isolating the contribution of premotor signals to SoA. The performance of active actions is accompanied by the generation of motor predictions (i.e., expectations about the sensory feedback produced by one’s own action) and volitional signals (i.e., intention-to-move). These premotor signals are produced only with voluntary actions^31^ and make an important internal cue to SoA during active movements as compared to passive ones. In line with this hypothesis, a recent study showed that the activation of premotor cortices at the stage of planning is crucial for the manifestation of the ‘feeling of agency’ during active as compared to passive movements^32^. The ‘*feeling of agency*’ is the immediate non-conceptual feeling of being the author of the movement^26,33^, and can be further processed to form a SoA judgement^33^. According to a generally accepted theoretical account, one of the more important mechanisms contributing to the feeling of agency is the comparator mechanism. This mechanism compares the motor prediction and the actual sensory feedback of a self-generated action. When the sensory reafference is congruent with motor prediction, the sensory feedback is labelled as self-generated^26^ and feeling of agency can arise over that action and its sensory consequences.

Compared to controls, patients’ SoA showed a less marked difference between active and passive conditions, meaning that patients may subjectively experience more similar intensities of SoA during the two movement modes. A dysfunction in action-related predictive signaling has been suggested in schizophrenia^29,34–36^ and it may explain patients’ SoA modulation in the present study. In other terms, an impairment to the detriment of motor predictions would make patients less able to distinguish whether an action is internally generated or not, leading them to provide SoA judgments with more similar scores between active and passive movements. In addition, a recent study investigated neural correlates of self-action recognition based on both the processing of movement mode and hand identity (one’s own vs. other hand) in schizophrenia. Results indicated that in healthy participants the activation of the angular gyrus is significantly modulated by both cues of self-other distinction. However, the activation of the angular gyrus in patients was significantly modulated by hand identity only, supporting the hypothesis that dysfunctional motor predictions render these sensorimotor cues unreliable in schizophrenia patients^37^.

In brief, the modulation of patients’ SoA is less strongly dependent on the type of movement. This result suggests that movement mode makes a less strong cue to SoA in schizophrenia. Current finding may be accounted by impairments in basic sensorimotor mechanisms activated by the generation of voluntary actions and that underlie the arising of the feeling of agency (but consider also other potential cognitive impairments discussed below).

It is worth highlighting that SoO and SoA judgments can be determined by factors that are independent from any sensorimotor process at the most basic level of action control and action perception^26,33^. As we used explicit judgments of SoO and SoA to investigate embodiment, we cannot infer whether the abnormal modulation of patients’ judgments is caused by deficits involving the pre-reflective level of SoA and SoO or by impairments in the conceptual level of them. On the one hand, the attenuated modulation of patients’ SoO and SoA may be due to aberrancies affecting the processing of perceptual and sensorimotor information arising during active and passive actions. Impairments like those discussed in previous paragraphs pertain to the pre-conceptual level of SoO and SoA. On the other hand, our results may be due to impairment in the processing of cognitive cues to SoO and SoA. In this regard, we cannot rule out the effect of higher-order causal inferences that may be anomalous in patients. For instance, patients may have been prone to provide similar scores of SoO irrespective to experimental manipulations because of particular attitude towards particular body sensations, e.g. phenomena of somatic depersonalization (e.g. “*I have the strange feeling that it’s somebody else’s body*”^38^). Similarly, patients may have rated SoA sensations similarly across conditions because of the anomalous interpretation of contextual agency cues^33^. For example, some patients may have been prone to assume the experimenters were always in control of the lever, thus ignoring their internal agency cues to judge SoA items. In addition, the role of aberrant metacognitive mechanisms serving self-other distinction in schizophrenia has been stressed in recent literature. For instance, a recent study examined SoA in psychosis asking participants to judge whether the movement of a virtual hand was identical to their actual movement or distorted. Psychotic patients tended to erroneously self-attribute the movement at larger distortions than healthy participants. Moreover, patients showed an increased confidence in their SoA judgements, despite their reduced sensitivity to sensorimotor conflicts. This finding suggested a link between anomalous SoA judgments and poor metacognitive insight into the impairment in self-generated action recognition^2^. Deficits in metacognition for SoA judgments has been also found in nonpsychotic young individuals with a genetic propensity for schizophrenia spectrum disorders (22q11 deletion syndrome), suggesting that these metacognitive dysfunctions could be present before the onset of a full-blown psychosis^39^.

In the present study, we also administered control items that were similar to the illusion-specific items, but do not capture the phenomenological experience of SoO and SoA. They served as controls for task compliancy, suggestibility or expectancy^6^. Because these items were designed to assess unlikely embodiment sensations, we did not expect any significant effect of modulation. While SoO-control confirmed our hypothesis, SoA-control did not. Our results indicated that SoA-control sensations were modulated by movement mode and visuomotor delay. These effects were significantly stronger in controls than patients. This unexpected result may be due to the arrangement of our MBI experimental setup, which is partially different from that of moving RHI studies using similar control statements^6,8^. In the present experiment, half of SoA-control items assessed how much the subject had the illusory feeling that his own real hand was controlled by the mirrored hand (see items 13–14). Participants may have experienced these sensations during passive conditions because they were looking at somebody else hand moving on a lever akin to the lever they were resting on. The hand in the mirror was a real hand, which is different from the artificial hand used in moving RHI studies. Differently from a fake hand, a real hand can be the source for one’s own passive movements. For this reason, we suggest that SoA-control items in the present MBI experiment assessed likely sensations of external attribution of agency more than compliancy or suggestibility. Similar to results about SoA, the modulation of patients’ SoA-control scores is less significantly driven by experimental manipulations at play, in agreement with the hypothesis that they represent a less strong cue for agency attribution (i.e., Self- vs. Other- attribution of authorship) in patients.

Since passivity experiences are clinically relevant manifestations of SoA disturbances in schizophrenia^40^, e.g. delusions of control, we hypothesized a significant moderation effect of passivity severity on SoA judgments. Particularly, we expected that patients with a more severe passivity profile showed an even smaller modulation of SoA judgments by both experimental factors. However, analyses did not reveal any significant effect of passivity on SoA scores.

On the other hand, we found that the passivity severity significantly interacted with movement mode with respect to SoA-control scores. This unexpected result might indicate that patients with more severe passivity symptoms have an even greater difficulty rating sensations regarding external attribution of agency based on differences in movement mode. Nonetheless, this analysis may be affected by a little variation in the passivity symptoms in this cohort as well as by the non-normality of residuals, making the interpretation inconclusive. Therefore, a link between passivity symptoms and body SoA attribution cannot be excluded^21,22,41^ but it deserves additional investigation in future embodiment studies.

Lastly, we exploratorily investigated the potential effect of medication on the modulation of patients’ judgments. We found that patients receiving higher doses of neuroleptics rated SoO items with higher (more positive) scores irrespective to experimental manipulations. This result is puzzling and not straightforward to interpret. Other factors we did not control for might play a role in the relationship between SoO judgments and antipsychotic doses. Therefore, we refrain from drawing conclusions from this finding.

The present study has some limitations. First, no objective measures of ownership and agency were collected during the illusion. They may have helped discriminating whether the anomalous modulation of SoO and SoA judgments was mainly due to low-level sensorimotor deficits or to cognitive aberrancies.

Second, the “as-if” hypothetical structure of the questionnaire items might have been difficult to understand by some patients given that language comprehension deficits have been demonstrated in schizophrenia^42^. To limit comprehension difficulties, items were read aloud by the experimenter (MR) and explained to the participant if he showed difficulty understanding the sentence. To further explore this aspect, we controlled whether patients had a higher proneness than controls to disengage from understanding questionnaire items by analysing intra-individual variability of responses. Patients did not significantly differ from controls in this regard (${t}_{59}=1.499, p= .139$).

Third, we included some left-handers in our sample (5 controls and 3 patients). To verify the potential influence of hand dominance on results, we reran analyses including the effect of compatibility in the models. Compatibility refers to whether the hand subjected to illusion (the hand the participant placed behind the mirror) was participant’s dominant hand or not. Compatibility effect did not survive model selection for any questionnaire component, suggesting that participants’ handedness did not significantly influence the results of the present study.

Fourth, groups were not balanced for age. We thus explored whether age had an impact on subjects’ ratings. Age showed a significant moderation effect on Delay as regards SoO-control items in the schizophrenia group. No other relevant influences of age were found.

Fifth, the unbalanced proportion of male and female participants in the two groups, we also controlled for an effect of sex. The inclusion of this factor as fixed effect in the analyses indeed suggests that it impacts on SoA results as concerns the *Mode* × *Group* interaction.

To conclude, we investigated SoO and SoA in schizophrenia by means of a visuomotor body illusion, that is the MBI. Our findings suggest that the type of movement to be performed (active vs. passive) and the degree of temporal congruence between visuomotor signals represent less salient cues to SoO and SoA in patients. Passivity symptoms might play a role in the processes underlying the recognition of the source of one’s own body sensations (Self vs. Other), however this result warrants future research.

## Methods

### Participants

Thirty-two participants with DSM-IV-TR diagnosis of schizophrenia were recruited from the Bolzano Hospital Mental Health department, Milan Santi Paolo e Carlo Hospital Mental Health Department and their community services. All patients were on antipsychotic medication. Three patients were excluded from the final analyses. One patient had difficulties understanding items phrased in Italian. Two patients took part in similar embodiment studies, and they were thus excluded to avoid their expectations biased results. The *Scale for the Assessment of Negative Symptoms* and the *Scale for the Assessment of Positive Symptoms*—SANS/SAPS^43,44^ were used to assess psychopathology. Thirty-two healthy participants were involved in the study through word of mouth or through the online recruitment system of the Department of Psychology (University of Milano-Bicocca). Students received course credits for their participation. See Table 1 for sample demographic and clinical information.

**Table 1:** Demographics and clinical characteristics of the sample at the time of testing.

	Patients	Controls
N	29	32
Gender, female, n	8	20
Age, mean ± SD (year)	44.31 ± 12.19	26.72 ± 9.77
Educational level, mean ± SD (year)	11.66 ± 2.96	16.88 ± 2.31
Handedness, right	26	27
Time since diagnosis, mean ± SD (year)	18 ± 9.93	–
**SANS**		
Affective flattening, mean ± SD	2.86 ± 1.42	–
Alogia, mean ± SD	2.74 ± 1.31	–
Avolition-Apathy, mean ± SD	3.16 ± 1.35	–
Anhedonia-Asociality, mean ± SD	3.34 ± 1.33	–
Attention, mean ± SD	3.2 ± 1.03	–
**SAPS**		
Hallucinations, mean ± SD	1.08 ± 1.14	–
Delusions, mean ± SD	1.97 ± 1.2	–
Bizarre behavior, mean ± SD	2.19 ± 1.52	–
Formal thought disorder, mean ± SD	1.99 ± 1.08	–
**Neuroleptics**		
First generation, n	3	–
Second generation, n	26	–
OLZ equivalents, mean ± SD (mg/day)	15.89 ± 7.85	–

The experimental protocol was approved by the Ethical Committees of the University of Milano-Bicocca and Bolzano Hospital. The study was carried out in accordance with the principles of the Declaration of Helsinki (World Medical Organization, 1996). All participants signed written informed consent. All participants were naïve to the hypothesis of the study and were debriefed at the end of the experiment.

### Experimental design and procedure

The experiment has a 2 × 4 full factorial design: *Mode of Movement* (Active, Passive) × *Delay* (120, 220, 320, 420 ms), 8 conditions overall. The procedure was divided in two sessions (Active and Passive session), each one lasting around 25–30 min, with around 10-min pause between them to reduce fatigue and/or boredom.

**Figure 3:**
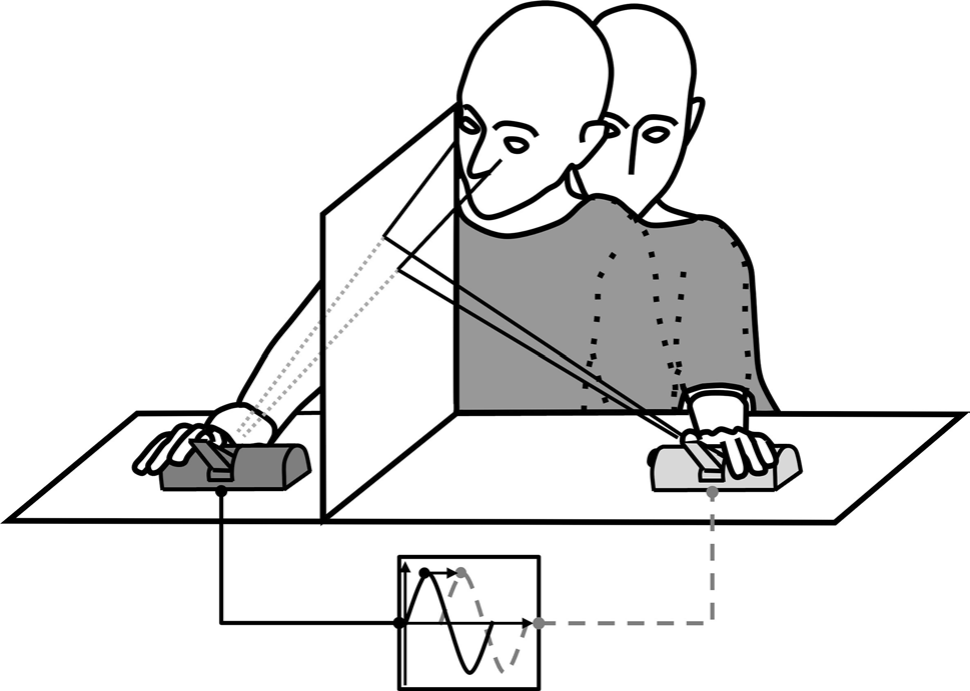
Experimental setup. The schematic diagram depicts the arrangement of the experimental setup when the hand subjected to the illusion was the right one. A mirror in sagittal orientation with respect to participant was placed on the table. The participant positioned his right hand behind the mirror over the lever (the master lever in case of active condition and the slave lever in case of passive condition). During each experimental condition the experimenter placed his (left) hand on the lever in front of the reflective surface. Therefore, the participant could see the reflection of experimenter’s hand spatially superimposed to his own hand when he looked toward it. We used cardboard boxed (not depicted in the figure) to limit the appearance of the experimenter’s hand on the participant’s (left) visual hemifield during the MBI induction phase. Each lever was made with a plastic bar that could move 30 degrees about a fixed pivot. By an electronic control system, delays of different duration could be applied on the onset of actuation of the rotor controlling the slave lever.

During each condition, the participant was required to press a lever with his hand positioned behind a mirror while looking at the reflective surface. The mirror was aligned with the participant’s midsagittal plane, mounted at the center of and perpendicular to the table. The experimenter (IR) placed her hand in front of the reflective surface on a lever identical to the one used by the participant. In this way, the participant could see the reflection of the experiment’s hand visually superimposed to his actual hand when pointing his gaze towards the location of this latter. To manipulate movement mode and visuomotor synchrony, we used an electromechanical device consisting of two levers, i.e., the master lever and the slave lever. The master lever controlled the actuation of the slave lever, meaning that when the master lever was pushed, its movement was transmitted to the slave lever that replicated the rotation (intrinsic delay of the apparatus: 40 ± 20 ms). The participant actively pressed the master lever during the active session, whereas he was to rest his finger onto the slave lever during the passive session (Fig. 3). Four different delays were imposed between the onset of actuation of the two levers to manipulate visuomotor synchrony. Each MBI induction phase lasted 1 min. The order of visuomotor delay presentation was counterbalanced between subjects. In each group, half of the participants were tested on the right hand, while the other half on the left hand.

16-item questionnaire was administered immediately after each MBI phase to assess subjective embodiment sensations. Statements were translated in Italian from the questionnaire used by Kalckert and Ehrsson^6^. Half of the items focused on SoO and SoA, the other half consisted in control statements (SoO-control, SoA-control) which were used to probe the occurrence of unlikely experiences of SoO and SoA. Participants rated each item on a 7-point Likert scale shown to the participant on a sheet of paper (+ 3: strong agreement; 0: neither agreement nor disagreement; −3: strong disagreement). To avoid any systematic response pattern (e.g., some patients may be prone to fixate on extreme points of the scale), participants were strongly encouraged to consider all the seven possible answers.

In order participants familiarized with correctly pushing the lever, they were invited to practice the movement at the very beginning of the active session. They were also asked to keep the forearm of the limb under test as still as possible during every 1-min MBI induction phase. To limit the rubbing of the fingertip against the surface of the lever, the pad was attached to the lever surface by a little piece of double-sided adhesive tape. The study was carried out by two experimenters. An experimenter (IR, female hand) played the role of the “mirrored hand”, while the other one (MR) coordinated the whole experimental procedure administering the questionnaire and giving instructions.

### Data analysis

Separate analyses were carried out for each questionnaire component, namely SoO, SoO-control, SoA, and SoA-control. Average scores (e.g., the mean of scores provided by the participant to the four items of SoO) were used as dependent variable. Data were fitted with linear mixed models that specifies the fixed effects of *Mode of Movement, Delay* and *Group*, the by-subject random intercept, and the heterogeneous residual variance between the two groups of participants (*see* Supplementary Materials for additional information about mixed model-building procedure and model diagnostics).

To test for an association between current episodes of passivity and embodiment sensations, we fitted patients’ mean scores with an index of passivity profile severity, i.e. sum of the ratings received by patients to 15–19 SAPS items^22,25,29,45–47^ as fixed effect together with *Mode of Movement* and *Delay.* Analogous analyses were conducted to test in an exploratory way the influence of antipsychotic treatment expressed in olanzapine-equivalent values for daily dose of neuroleptic medication^48^.

To explore possible associations between SoO, SoA and SAPS/SANS subscales, we also run correlations in each group separately.

## Supplementary Information

Supplementary Information 1.

Supplementary Information 2.

## Data Availability

Raw questionnaire data are made available as Supplementary dataset.

## References

[1] Lenggenhager, B. & Lopez, C. Vestibular contributions to the sense of body, self, and others. *Lenggenhager, Bigna; Lopez, Christophe (2015). Vestib. Contrib. to Sense Body, Self, Others. Metzinger, Thomas; Wind. Jennifer M. Open MIND. Frankfurt am Main MIND Group, 1–38.* (2015). doi:10.5167/uzh-139675.

[2] Krugwasser, A. R., Stern, Y., Faivre, N., Harel, E. V., Salomon, R. Impaired sense of agency and associated confidence in psychosis. *Schizophrenia* **8**, 1–8 (2022). 10.1038/s41537-022-00212-4 PMC9261084 35854004

[3] Botvinick M, Cohen J (1998). Rubber hands’ feel’touch that eyes see. Nature.

[4] Kilteni K, Maselli A, Kording KP, Slater M (2015). Over my fake body: body ownership illusions for studying the multisensory basis of own-body perception. Front. Hum. Neurosci..

[5] Azanõn E (2016). Multimodal contributions to body representation. Multisens. Res..

[6] Kalckert A, Ehrsson HH (2012). Moving a rubber hand that feels like your own: a dissociation of ownership and agency. Front. Hum. Neurosci..

[7] Kalckert, A. & H. Ehrsson, H. The onset time of the ownership sensation in the moving rubber hand illusion. *Front. Psychol.* (2017). doi:10.3389/fpsyg.2017.00344. 10.3389/fpsyg.2017.00344 PMC5345084 28344566

[8] Kalckert A, Ehrsson HH (2014). The moving rubber hand illusion revisited: comparing movements and visuotactile stimulation to induce illusory ownership. Conscious. Cognit..

[9] Gallagher, S. Self-reference and schizophrenia: a cognitive model of immunity to error through misidentification. In *Exploring the Self: Philosophical and Psychopathological Perspectives on Self-experience* (2000).

[10] Braun N (2018). The senses of agency and ownership: a review. Front. Psychol..

[11] Hur, J. W., Kwon, J. S., Lee, T. Y. & Park, S. The crisis of minimal self-awareness in schizophrenia: a meta-analytic review. *Schizophr. Res.* **152**, (2014). 10.1016/j.schres.2013.08.042 24055201

[12] Peled A, Ritsner M, Hirschmann S, Geva AB, Modai I (2000). Touch feel illusion in schizophrenic patients. Biol. Psychiatry.

[13] Peled A, Pressman A, Geva AB, Modai I (2003). Somatosensory evoked potentials during a rubber-hand illusion in schizophrenia. Schizophr. Res..

[14] Thakkar KN, Nichols HS, McIntosh LG, Park S (2011). Disturbances in body ownership in schizophrenia: evidence from the rubber hand illusion and case study of a spontaneous out-of-body experience. PLoS ONE.

[15] Graham KT, Martin-Iverson MT, Holmes NP, Jablensky A, Waters F (2014). Deficits in agency in schizophrenia, and additional deficits in body image, body schema, and internal timing, in passivity symptoms. Front. Psychiatry.

[16] Ferri F (2014). Upcoming tactile events and body ownership in schizophrenia. Schizophr. Res..

[17] Shaqiri A (2018). Rethinking body ownership in schizophrenia: experimental and meta-analytical approaches show no evidence for deficits. Schizophr. Bull..

[18] Klaver M, Dijkerman HC (2016). Bodily experience in schizophrenia: factors underlying a disturbed sense of body ownership. Front. Hum. Neurosci..

[19] Postmes L (2014). Schizophrenia as a self-disorder due to perceptual incoherence. Schizophr. Res..

[20] Noel JP, Cascio CJ, Wallace MT, Park S (2017). The spatial self in schizophrenia and autism spectrum disorder. Schizophr. Res..

[21] Graham-Schmidt KT, Martin-Iverson MT, Waters FAV (2018). Self- and other-agency in people with passivity (first rank) symptoms in schizophrenia. Schizophr. Res..

[22] Laurin, A. *et al.* Self-consciousness impairments in schizophrenia with and without first rank symptoms using the moving rubber hand illusion. *Conscious. Cognit.* **93**, (2021). 10.1016/j.concog.2021.103154 34052640

[23] Rossetti I (2019). Defective embodiment of Alien hand uncovers altered sensorimotor integration in schizophrenia. Schizophr. Bull..

[24] Fletcher, P. C. & Frith, C. D. Perceiving is believing: a Bayesian approach to explaining the positive symptoms of schizophrenia. *Nat. Rev. Neurosci.* **10**, (2009). 10.1038/nrn2536 19050712

[25] Franck N (2001). Defective recognition of one’s own actions in patients with schizophrenia. Am. J. Psychiatry.

[26] Synofzik M, Vosgerau G, Newen A (2008). I move, therefore I am: A new theoretical framework to investigate agency and ownership. Conscious. Cognit..

[27] Ismail MAF, Shimada S (2016). ‘Robot’ hand illusion under delayed visual feedback: relationship between the senses of ownership and agency. PLoS ONE.

[28] Zhou, H. yu *et al.* Multisensory temporal binding window in autism spectrum disorders and schizophrenia spectrum disorders: a systematic review and meta-analysis. *Neurosci. Biobehav. Rev.* (2018). doi:10.1016/j.neubiorev.2017.12.013. 10.1016/j.neubiorev.2017.12.013 29317216

[29] Synofzik M, Thier P, Leube DT, Schlotterbeck P, Lindner A (2010). Misattributions of agency in schizophrenia are based on imprecise predictions about the sensory consequences of one’s actions. Brain.

[30] Franck, N., Posada, A., Pichon, S. & Haggard, P. Altered subjective time of events in schizophrenia. *J. Nerv. Ment. Dis.* **193**, (2005). 10.1097/01.nmd.0000161699.76032.09 15870620

[31] Farrer, C., Valentin, G. & Hupé, J. M. The time windows of the sense of agency. *Conscious. Cognit.* **22**, (2013). 10.1016/j.concog.2013.09.010 24161792

[32] Zapparoli L (2020). How the effects of actions become our own. Sci. Adv..

[33] Synofzik M, Vosgerau G, Newen A (2008). Beyond the comparator model: A multifactorial two-step account of agency. Conscious. Cognit..

[34] Leptourgos, P. & Corlett, P. R. Embodied predictions, agency, and psychosis. *Front. Big Data* **3**, (2020). 10.3389/fdata.2020.00027 PMC7931869 33693400

[35] Sterzer, P. *et al.* The predictive coding account of psychosis. *Biol. Psychiatry* 634–643 (2018). doi:10.1016/j.biopsych.2018.05.015. 10.1016/j.biopsych.2018.05.015 PMC6169400 30007575

[36] van der Weiden A, Prikken M, van Haren NEM (2015). Self-other integration and distinction in schizophrenia: a theoretical analysis and a review of the evidence. Neurosci. Biobehav. Rev..

[37] Uhlmann, L., Pazen, M., Van Kemenade, B. M., Kircher, T. & Straube, B. Neural correlates of self-other distinction in patients with schizophrenia spectrum disorders: the roles of agency and hand identity. *Schizophrenia Bull.* **47**, (2021). 10.1093/schbul/sbaa186 PMC8379550 33433625

[38] Parnas J (2005). EASE: examination of anomalous self-experience. Psychopathology.

[39] Salomon, R. *et al.* Agency deficits in a human genetic model of schizophrenia: insights from 22q11DS patients. *Schizophr. Bull.* **48**, (2022). 10.1093/schbul/sbab143 PMC8886583 34935960

[40] Frith C (2012). Explaining delusions of control: the comparator model 20years on. Conscious. Cognit..

[41] Blakemore SJ, Smith J, Steel R, Johnstone EC, Frith CD (2000). The perception of self-produced sensory stimuli in patients with auditory hallucinations and passivity experiences: evidence for a breakdown in self-monitoring. Psychol. Med..

[42] Boudewyn, M. A., Carter, C. S. & Swaab, T. Y. Cognitive control and discourse comprehension in schizophrenia. *Schizophr. Res. Treatment* **2012**, (2012). 10.1155/2012/484502 PMC3420642 22970364

[43] Andreasen, N. C. Scale for the assessment of negative symptoms (SANS). *Br. J. Psychiatry* (1989). 2695141

[44] Andreasen N (1984). The scale for the assessment of positive symptoms (SAPS). Univ. Iowa.

[45] Fourneret P (2002). Perception of self-generated action in schizophrenia. Cognit. Neuropsychiatry.

[46] Daprati E (1997). Looking for the agent: an investigation into consciousness of action and self-consciousness in schizophrenic patients. Cognition.

[47] Fourneret P, Franck N, Slachevsky A, Jeannerod M (2001). Self-monitoring in schizophrenia revisited. NeuroReport.

[48] Gardner, D. M., Murphy, A. L., O’Donnell, H., Centorrino, F. & Baldessarini, R. J. International consensus study of antipsychotic dosing. *Am. J. Psychiatry* **167**, (2010). 10.1176/appi.ajp.2009.09060802 20360319

