# Supplementary material for: Sense of body ownership and body agency in schizophrenia
Source: Sci Rep. 2022 Nov 12;12:19369. doi: 10.1038/s41598-022-23988-y (PMC9653492; doi:10.1038/s41598-022-23988-y)
Supplement: Supplementary file 1 — Supplementary Information 1. [file 41598_2022_23988_MOESM1_ESM.pdf]

Sense of body ownership and body agency in schizophrenia.

Ileana Rossetti, Martina Repossi, Vincenzo Florio, Benedetta Demartini,
Andreas Conca, Orsola Gambini, Angelo Maravita

Supplementary information

S1 Specification of the statistical models and analyses

We analysed questionnaire data with LMM (lme {nlme}) in order to accommodate the repeated-measures experimental design. The analysis has been carried out employing procedures illustrated in West et al. (2007).

We started building a model including the fixed effects of all the covariates (and interactions) we wished to consider (*Mode of Movement*, *Delay* and *Group*) using Restricted Maximum Likelihood (REML) estimation of parameters. A second model also included by-subject random intercept. We compared these two models to confirm the need for the random predictor. Because a larger variability of data was apparent in patients' group according to summary statistics and boxplot in all questionnaire components, we explored whether to retain heterogeneous residual variance structures. In a third model we allowed residual variance parameters to differ between levels of the categorical variable *Group* and checked model fitting improvement. We next refitted the preferred model using Maximum Likelihood (ML) estimation of parameters and passed it to an automated reduction procedure based on the AIC-based selection of fixed-effect parameters (stepAIC {MASS}). We kept model resulting from this procedure as our final model. The REML-based final model was lastly used to test fixed effects (F-test, marginal SS) and to carry out diagnostics of residuals and random effects ({HLMdiag}).

Shown below boxplots and descriptive statistics of each embodiment component scores, the summary statistics of the final models, the output of the F-tests and model diagnostics. In the final model table, the upper part lists the fixed-effect parameter estimates, their standard error (SE), confidence interval and corresponding t-tests. The lower subsection shows random-effect statistics describing the random-intercept variance (σ^2), the variation between individual intercepts and average intercept ($\tau_{00\ \mathrm{ID}}$) and the intraclass correlation (ICC).

S1.1 SoO

Descriptive statistics

	Group	Mode	Delay	N	MEAN	SD	95%CI
1	CTR	Active	120	32	1.52	1.33	±0.48
2	CTR	Active	220	32	0.53	1.11	±0.4
3	CTR	Active	320	32	0.05	1.09	±0.39
4	CTR	Active	420	32	-0.21	0.90	±0.32
5	CTR	Passive	120	32	0.50	1.00	±0.36
6	CTR	Passive	220	32	-0.03	1.07	±0.39
7	CTR	Passive	320	32	-0.45	0.95	±0.34
8	CTR	Passive	420	32	-0.55	1.02	±0.37
9	SCZ	Active	120	29	0.55	1.34	±0.51
10	SCZ	Active	220	29	0.61	1.73	±0.66
11	SCZ	Active	320	29	0.33	1.30	±0.49
12	SCZ	Active	420	29	0.47	1.28	±0.49
13	SCZ	Passive	120	29	-0.37	1.68	±0.64
14	SCZ	Passive	220	29	0.07	1.18	±0.45
15	SCZ	Passive	320	29	-0.08	1.20	±0.46
16	SCZ	Passive	420	29	-0.22	1.17	±0.45

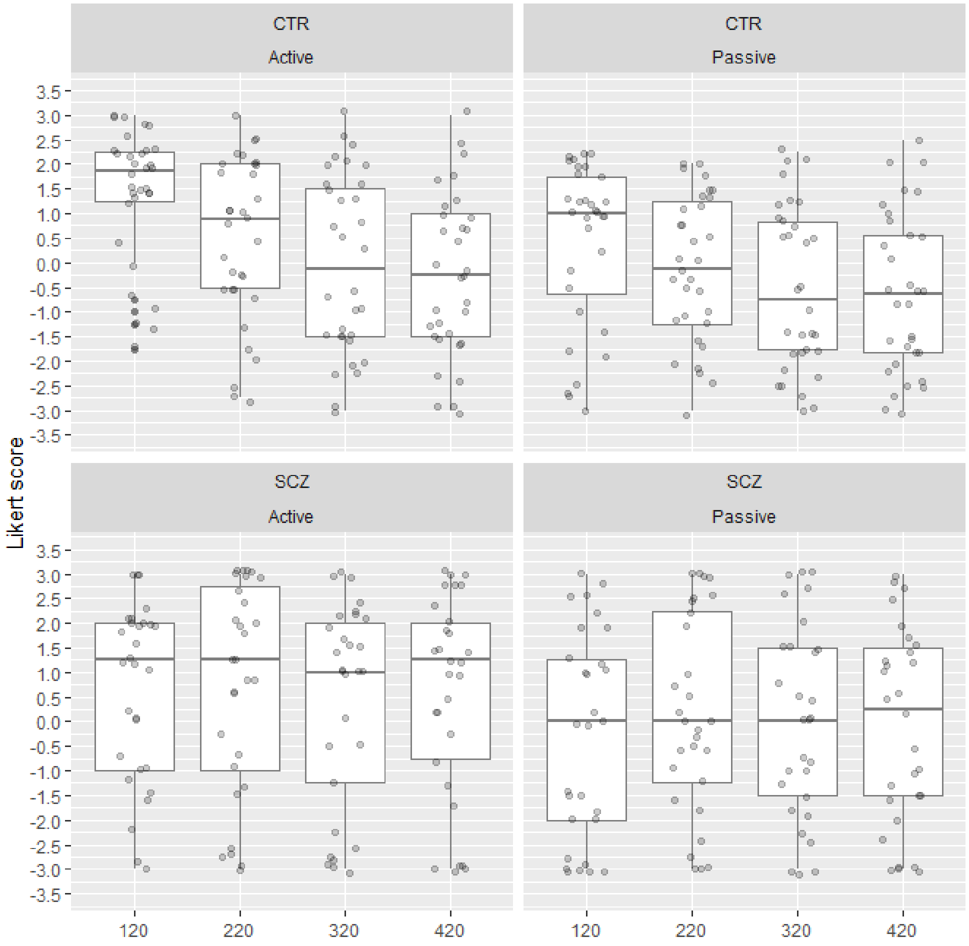

Final model

The final mixed model for SoO scores has fixed effects associated with *Mode of Movement*, *Delay, Group* and the *Delay*×*Group* interaction, a random intercept for each subject and separate residual variance parameters for the two groups of participants.

Ownership

	Score					
Predictors	*Estimates*	*SE*	*95%CI*	*t value*	*p*	*df*
(Intercept)	1.24	0.28	0.70 – 1.79	4.47	**<0.001**	420.00
Mode [Passive]	-0.62	0.11	-0.83 – -0.41	-5.77	**<0.001**	420.00
Delay [220]	-0.76	0.19	-1.13 – -0.39	-4.04	**<0.001**	420.00
Delay [320]	-1.21	0.19	-1.59 – -0.84	-6.44	**<0.001**	420.00
Delay [420]	-1.39	0.19	-1.76 – -1.02	-7.38	**<0.001**	59.00
Group [SCZ]	-0.76	0.41	-1.58 – 0.07	-1.84	0.071	420.00
Delay [220] * Group [SCZ]	1.01	0.32	0.39 – 1.63	3.20	**0.001**	420.00
Delay [320] * Group [SCZ]	1.25	0.32	0.63 – 1.87	3.95	**<0.001**	420.00
Delay [420] * Group [SCZ]	1.42	0.32	0.80 – 2.04	4.49	**<0.001**	420.00
**Random Effects**						
σ^2	1.14					
$\tau_{00\ ID}$	1.82					
ICC	0.61					
N_{ID}	61					
Observations	488					
AIC	1737.948					
log-Likelihood	-856.974					

Model diagnostics

Normality of random effects. Shapiro-Wilk test of normality (W = 0.97109, p-value = 0.1577).

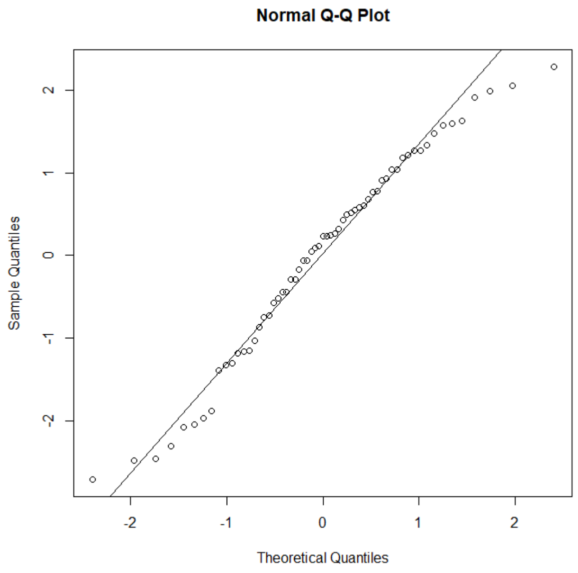

Normality of conditional raw residuals (pooled by Group). Shapiro-Wilk test of normality (Controls: W = 0.99533, p-value = 0.6332; Patients: W = 0.98903, p-value = 0.07447).

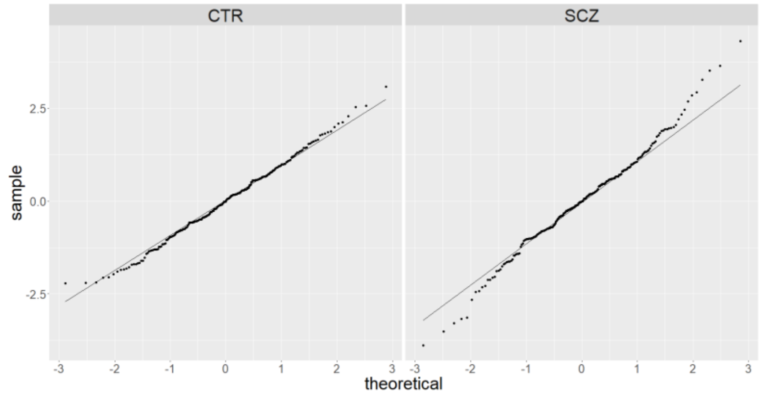

Homogeneity of variance of conditional raw residuals (pooled by Group). Levene's test (Controls: $F_{7,248} = 0.473, p = .854$; Patients: $F_{7,224} = 0.794, p = .594$).

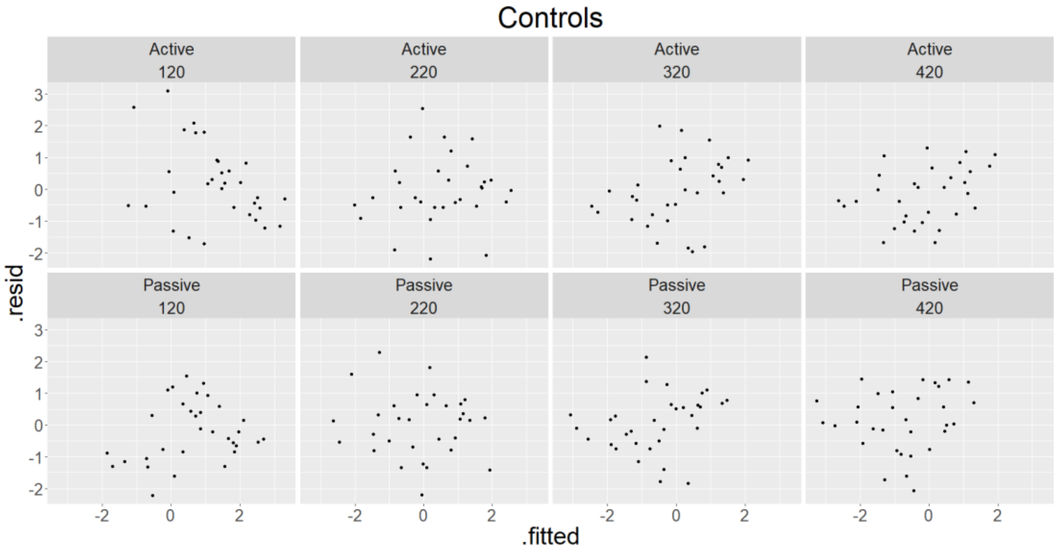

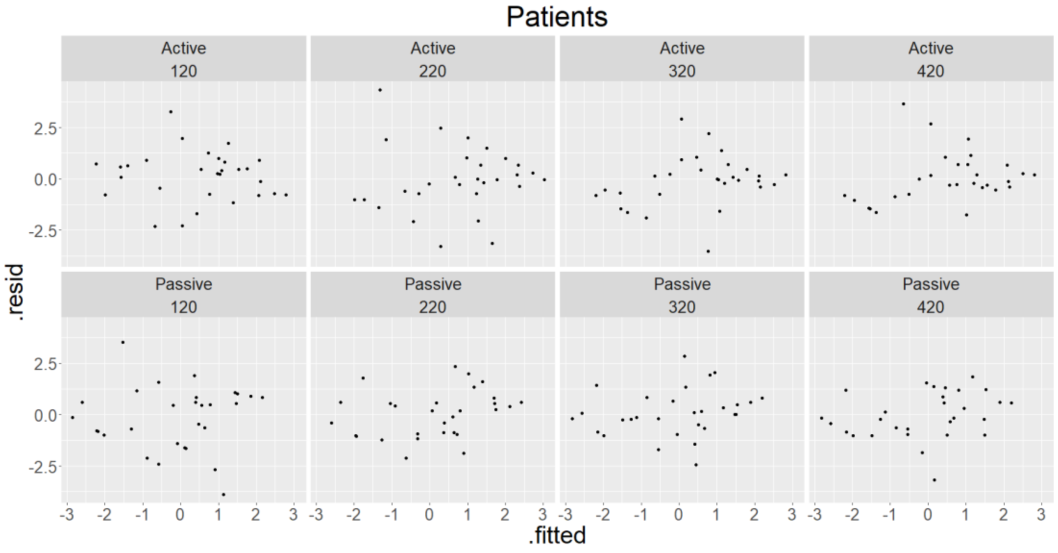

S1.2 SoA

Descriptive statistics

	Group	Mode	Delay	N	MEAN	SD	95%CI
1	CTR	Active	120	32	2.42	1.02	±0.37
2	CTR	Active	220	32	1.49	1.01	±0.36
3	CTR	Active	320	32	0.87	1.12	±0.4
4	CTR	Active	420	32	0.40	1.03	±0.37
5	CTR	Passive	120	32	-0.83	1.05	±0.38
6	CTR	Passive	220	32	-1.57	0.75	±0.27
7	CTR	Passive	320	32	-1.73	0.84	±0.3
8	CTR	Passive	420	32	-1.76	0.77	±0.28
9	SCZ	Active	120	29	1.54	1.55	±0.59
10	SCZ	Active	220	29	0.90	1.27	±0.48
11	SCZ	Active	320	29	0.59	1.04	±0.39
12	SCZ	Active	420	29	0.62	0.95	±0.36
13	SCZ	Passive	120	29	-0.74	1.17	±0.45
14	SCZ	Passive	220	29	-1.17	0.97	±0.37
15	SCZ	Passive	320	29	-1.20	1.22	±0.46
16	SCZ	Passive	420	29	-1.27	1.26	±0.48

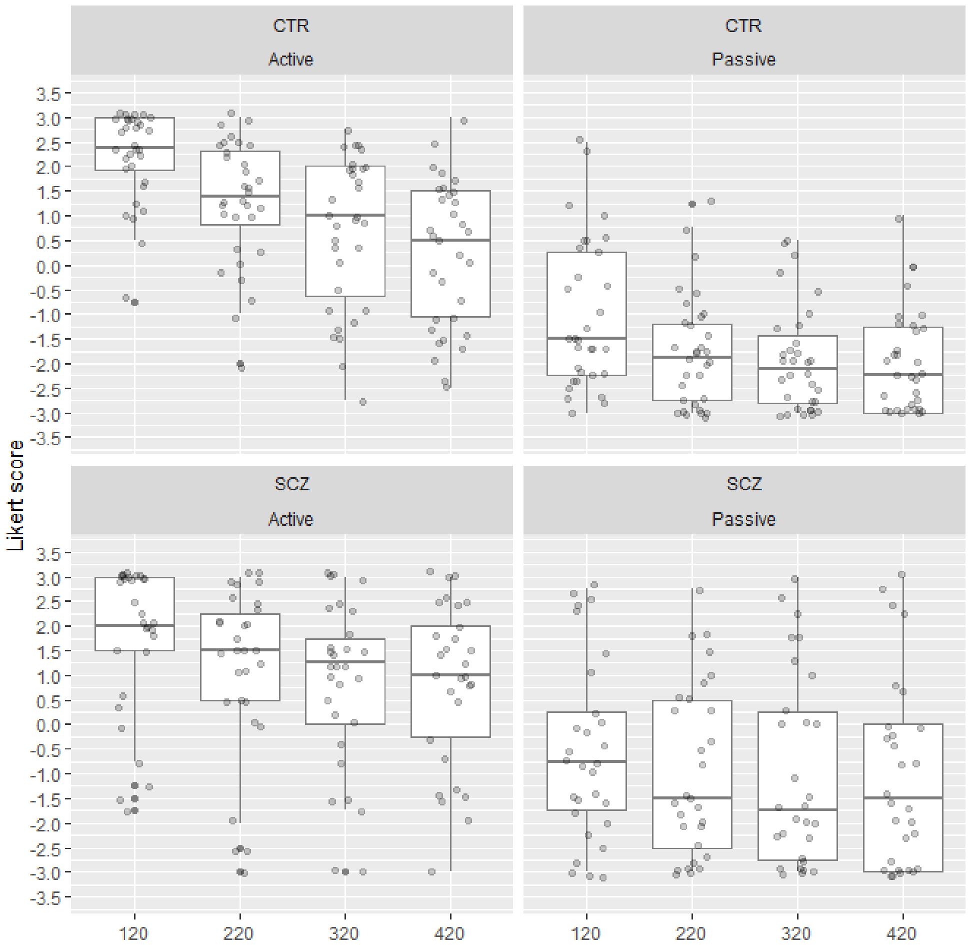

Final model

The final mixed model for SoA scores has fixed effects associated with *Mode of Movement*, *Delay, Group* and the interactions *Mode×Delay, Mode×Group, Delay×Group*, a random intercept for each subject and different residual variance parameters for the two groups.

Agency

	Score					
Predictors	*Estimates*	*SE*	*95%CI*	*t value*	*p*	*df*
(Intercept)	2.18	0.24	1.70 – 2.66	8.92	**<0.001**	416.00
Mode [Passive]	-3.17	0.20	-3.57 – -2.77	-15.61	**<0.001**	416.00
Delay [220]	-0.93	0.22	-1.35 – -0.50	-4.30	**<0.001**	416.00
Delay [320]	-1.52	0.22	-1.94 – -1.09	-7.03	**<0.001**	416.00
Delay [420]	-1.89	0.22	-2.31 – -1.46	-8.75	**<0.001**	59.00
Group [SCZ]	-0.36	0.35	-1.06 – 0.34	-1.02	0.312	416.00
Mode [Passive] * Delay [220]	0.20	0.27	-0.33 – 0.73	0.74	0.462	416.00
Mode [Passive] * Delay [320]	0.59	0.27	0.06 – 1.12	2.20	**0.028**	416.00
Mode [Passive] * Delay [420]	0.84	0.27	0.31 – 1.37	3.12	**0.002**	416.00
Mode [Passive] * Group [SCZ]	0.76	0.20	0.37 – 1.15	3.85	**<0.001**	416.00
Delay [220] * Group [SCZ]	0.30	0.28	-0.25 – 0.85	1.07	0.286	416.00
Delay [320] * Group [SCZ]	0.52	0.28	-0.03 – 1.07	1.86	0.063	416.00
Delay [420] * Group [SCZ]	0.74	0.28	0.20 – 1.29	2.67	**0.008**	416.00
**Random Effects**						
σ^2	0.91					
τ_{00} ID	1.13					
ICC	0.55					
N ID	61					
Observations	488					
AIC	1617.378					
log-Likelihood	-792.689					

Model diagnostics

<u>Normality of random effects</u>. Shapiro-Wilk test of normality (W = 0.98966, p-value = 0.8877).

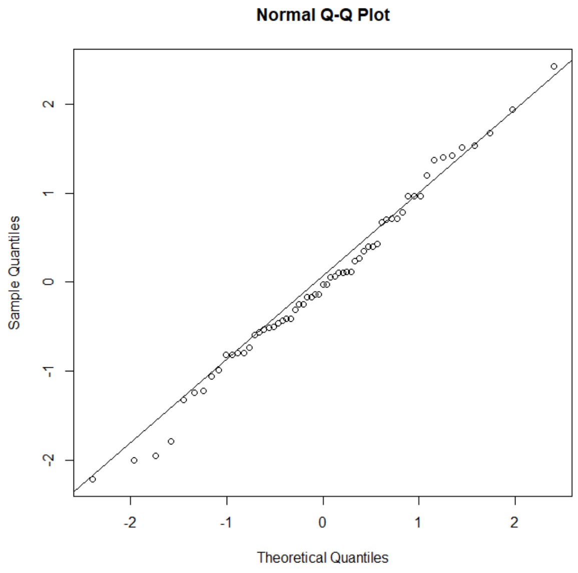

Normality of conditional raw residuals (pooled by Group). Shapiro-Wilk test of normality (Controls: W = 0.99259, p-value = 0.2304; Patients: W = 0.99352, p-value = 0.4126).

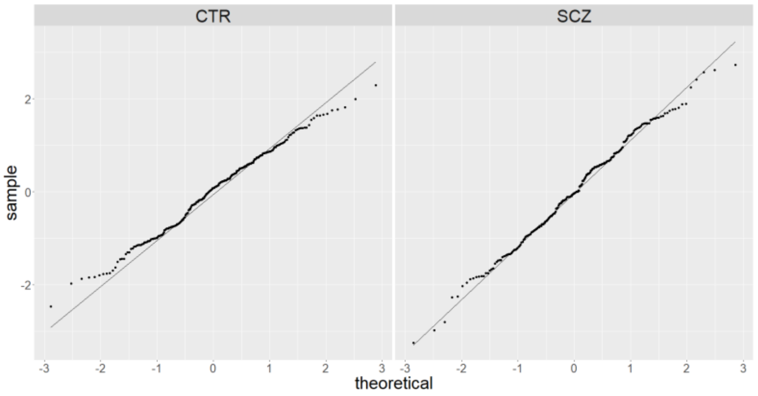

Homogeneity of variance of conditional raw residuals (pooled by Group). Levene's test (Controls: $F_{7,248} = 1.388, p = .211$; Patients: $F_{7,224} = 1.181, p = .315$).

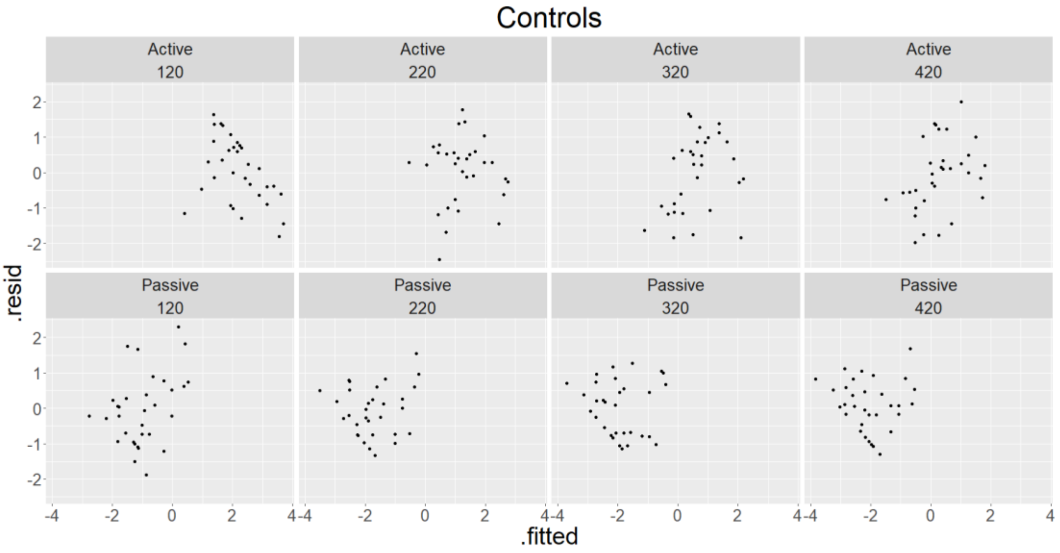

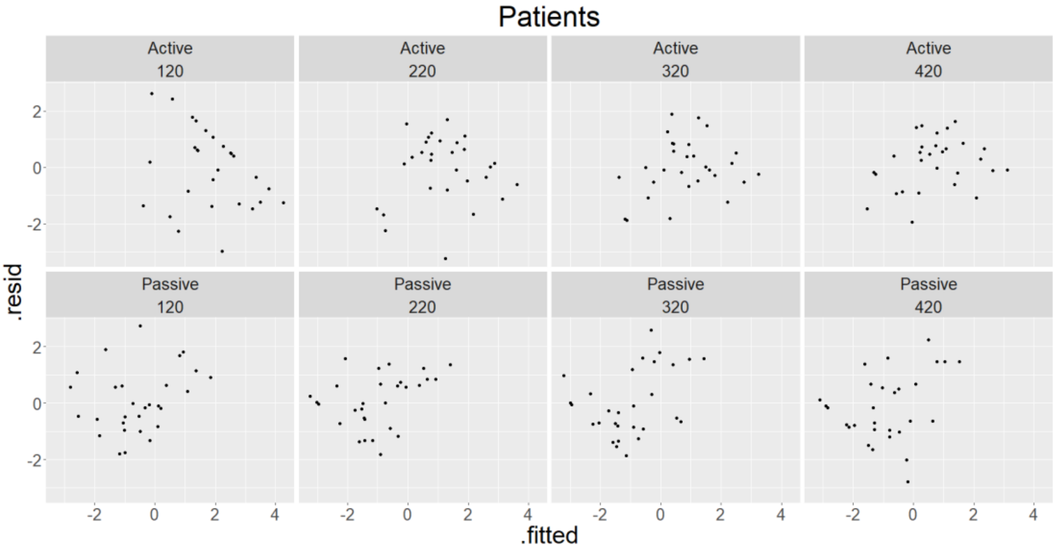

S1.3 SoO-control

Descriptive statistics

	Group	Mode	Delay	N	MEAN	SD	95%CI
1	CTR	Active	120	32	-1.43	0.76	±0.27
2	CTR	Active	220	32	-1.28	0.56	±0.2
3	CTR	Active	320	32	-1.36	0.62	±0.22
4	CTR	Active	420	32	-1.37	0.61	±0.22
5	CTR	Passive	120	32	-1.18	0.71	±0.25
6	CTR	Passive	220	32	-1.33	0.66	±0.24
7	CTR	Passive	320	32	-1.13	0.85	±0.31
8	CTR	Passive	420	32	-1.14	0.56	±0.2
9	SCZ	Active	120	29	-1.33	0.80	±0.31
10	SCZ	Active	220	29	-1.15	0.95	±0.36
11	SCZ	Active	320	29	-1.41	0.80	±0.3
12	SCZ	Active	420	29	-1.26	0.85	±0.32
13	SCZ	Passive	120	29	-1.60	0.87	±0.33
14	SCZ	Passive	220	29	-1.08	0.82	±0.31
15	SCZ	Passive	320	29	-0.94	0.92	±0.35
16	SCZ	Passive	420	29	-1.44	1.21	±0.46

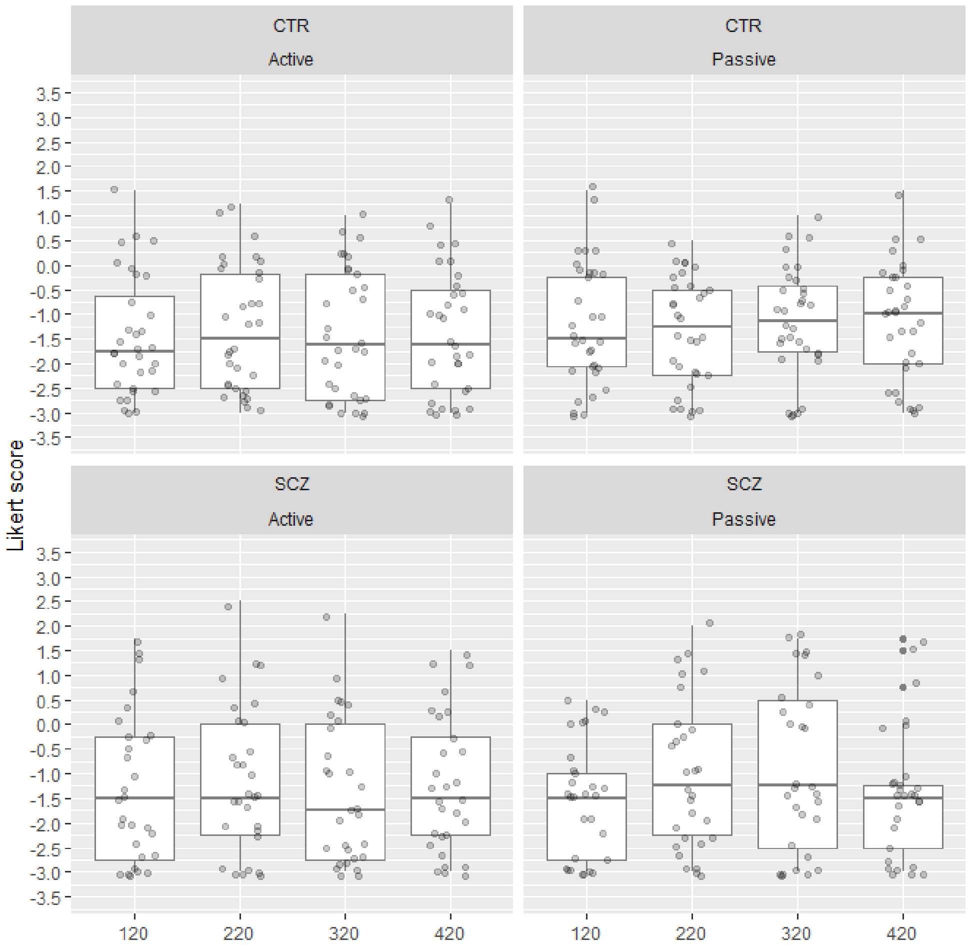

Final model

Model for SoO-control data includes a random intercept for each subject and separate residual variance parameters for the two groups of participants. The stepwise model selection has removed all fixed-effect parameters but the last effect *Mode of Movement.*

S1.3 SoA-control

Descriptive statistics

	Group	Mode	Delay	N	MEAN	SD	95%CI
1	CTR	Active	120	32	-1.67	0.92	±0.33
2	CTR	Active	220	32	-1.01	0.97	±0.35
3	CTR	Active	320	32	-0.88	0.93	±0.33
4	CTR	Active	420	32	-0.73	0.97	±0.35
5	CTR	Passive	120	32	0.81	1.00	±0.36
6	CTR	Passive	220	32	1.02	0.78	±0.28
7	CTR	Passive	320	32	1.17	0.80	±0.29
8	CTR	Passive	420	32	1.14	0.80	±0.29
9	SCZ	Active	120	29	-0.61	0.96	±0.37
10	SCZ	Active	220	29	-0.38	1.08	±0.41
11	SCZ	Active	320	29	-0.25	1.08	±0.41
12	SCZ	Active	420	29	-0.36	1.00	±0.38
13	SCZ	Passive	120	29	0.50	0.94	±0.36
14	SCZ	Passive	220	29	0.44	1.04	±0.39
15	SCZ	Passive	320	29	0.31	1.05	±0.4
16	SCZ	Passive	420	29	0.19	0.98	±0.37

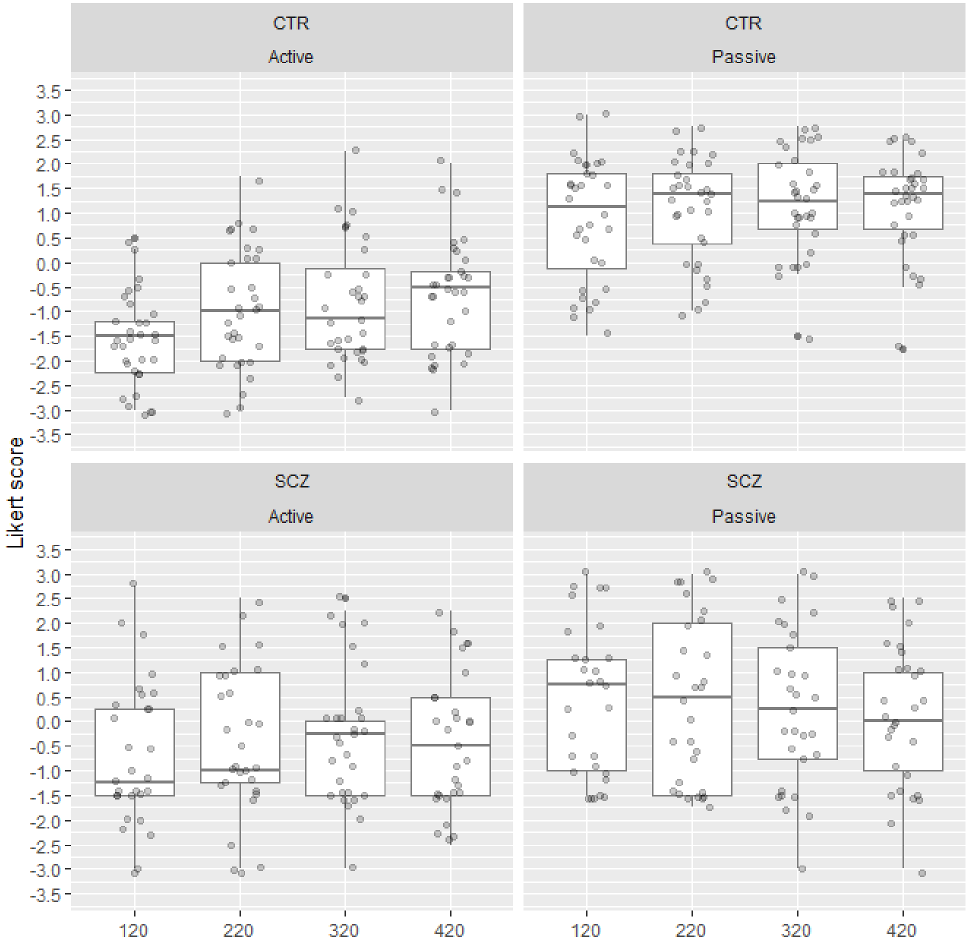

Final model

The final mixed model for SoA-control scores has fixed effects associated with *Mode of Movement*, *Delay, Group* and the interactions *Mode×Delay, Mode×Group, Delay×Group*, a random intercept for each subject and separate residual variance parameters for the two groups of participants.

Agency - Control items

	Score					
Predictors	*Estimates*	*SE*	*95%CI*	*t value*	*p*	*df*
(Intercept)	-1.60	0.21	-2.02 – -1.19	-7.53	**<0.001**	416.00
Mode [Passive]	2.47	0.19	2.10 – 2.83	13.27	**<0.001**	416.00
Delay [220]	0.62	0.20	0.23 – 1.01	3.13	**0.002**	416.00
Delay [320]	0.81	0.20	0.42 – 1.20	4.07	**<0.001**	416.00
Delay [420]	0.93	0.20	0.54 – 1.32	4.66	**<0.001**	59.00
Group [SCZ]	0.92	0.30	0.33 – 1.52	3.09	**0.003**	416.00
Mode [Passive] * Delay [220]	-0.38	0.24	-0.86 – 0.10	-1.56	0.118	416.00
Mode [Passive] * Delay [320]	-0.47	0.24	-0.95 – 0.00	-1.95	0.052	416.00
Mode [Passive] * Delay [420]	-0.59	0.24	-1.07 – -0.11	-2.43	**0.016**	416.00
Mode [Passive] * Group [SCZ]	-1.35	0.17	-1.69 – -1.00	-7.73	**<0.001**	416.00
Delay [220] * Group [SCZ]	-0.34	0.25	-0.83 – 0.14	-1.39	0.165	416.00
Delay [320] * Group [SCZ]	-0.49	0.25	-0.97 – -0.00	-1.98	**0.048**	416.00
Delay [420] * Group [SCZ]	-0.66	0.25	-1.15 – -0.18	-2.69	**0.007**	416.00
**Random Effects**						
σ^2	0.80					
$\tau_{00\ ID}$	0.78					
ICC	0.49					
N_{ID}	61					
Observations	488					
AIC	1504.136					
log-Likelihood	-736.068					

Model diagnostics

Normality of random effects. Shapiro-Wilk test of normality (W = 0.98671, p-value = 0.7494).

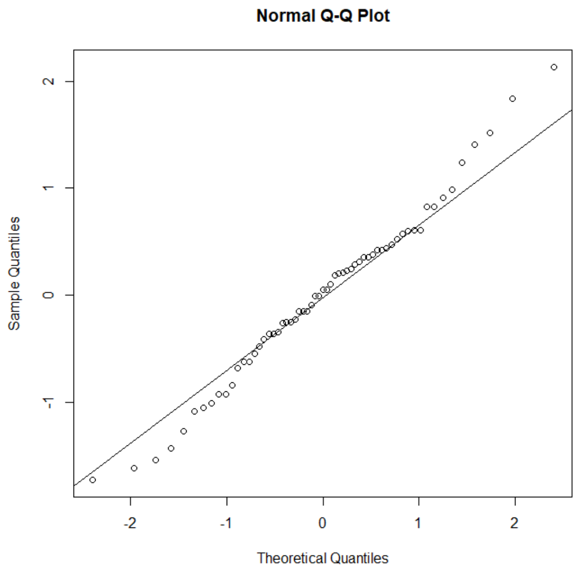

Normality of conditional raw residuals (pooled by Group). Shapiro-Wilk test of normality (Controls: W = 0.9947, p-value = 0.517; Patients: W = 0.9898, p-value = 0.1013)

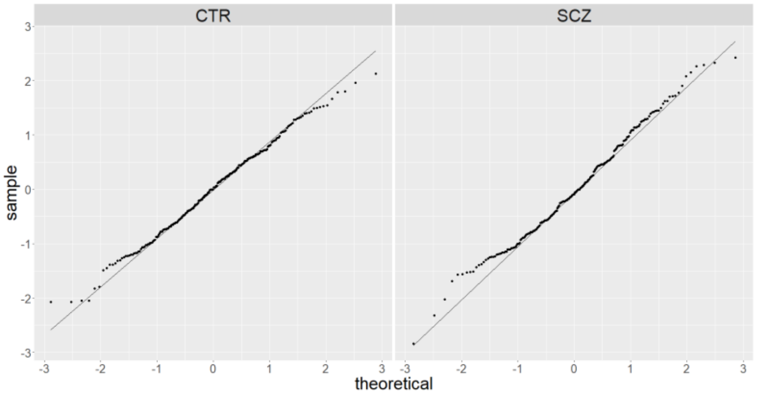

Homogeneity of variance of conditional raw residuals (pooled by Group). Levene's test (Controls: $F_{7,248} = 0.53, p = .812$; Patients: $F_{7,224} = .169, p = .991$).

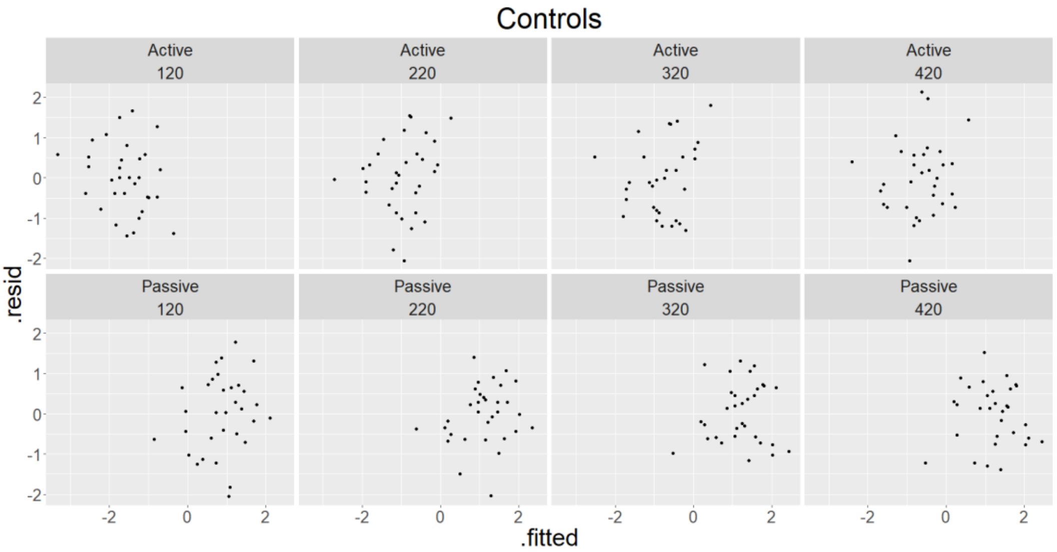

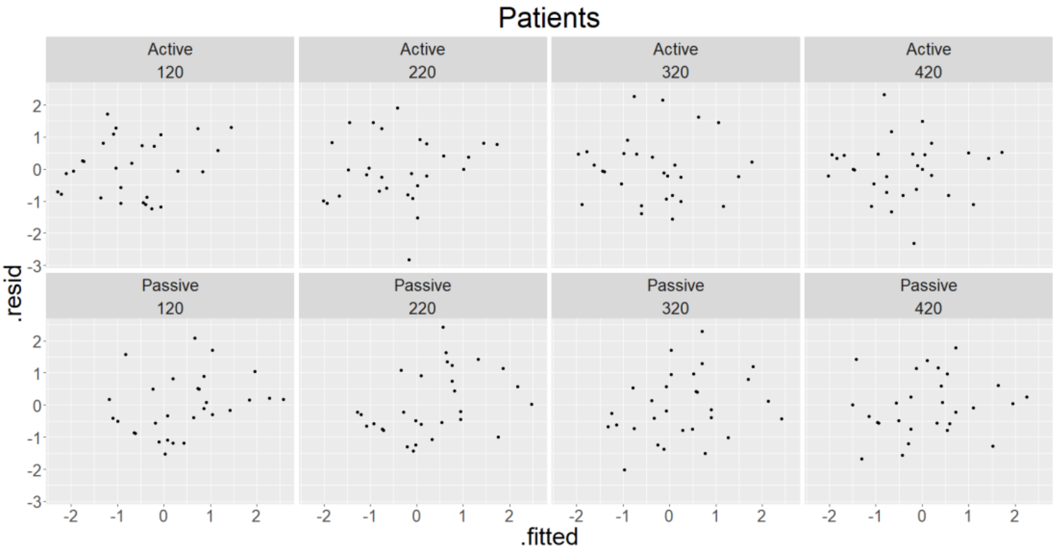

S2 Exploratory analyses on passivity symptom severity

S2.1 SoO

Final model

The final mixed model for patients' SoO data only retains the fixed effect of *Mode of Movement* in addition to the random intercept for each subject.

Model diagnostics

Normality of random effects. Shapiro-Wilk test of normality (W = 0.9581, p-value = 0.2948).

Normality of conditional raw residuals. Shapiro-Wilk test of normality (W = 0.9898, p-value = 0.0336).

Homogeneity of variance of conditional raw residuals. Levene's test ($F_{7,224} = .039, p = .843$).

S2.2 SoA

Final model

The final mixed model for patients' SoA data includes the fixed effect of *Mode of Movement* and *Delay* in addition to the random intercept for each subject.

Model diagnostics

Normality of random effects. Shapiro-Wilk test of normality (W = 0.9839, p-value = 0.9246).

Normality of conditional raw residuals. Shapiro-Wilk test of normality (W = 0.9951, p-value = 0.6638).

Homogeneity of variance of conditional raw residuals. Levene's test ($F_{7,224} = 1.327, p = .238$).

S2.3 SoO-Control

Final model

The final mixed model for patients' SoO data retains the fixed effect of *Mode of Movement, Passivity* and *Mode×Passivity* in addition to the random intercept for each subject.

Model diagnostics

Normality of random effects. Shapiro-Wilk test of normality (W = 0.9544, p-value = 0.2369).

Normality of conditional raw residuals. Shapiro-Wilk test of normality (W = 0.9782, p-value = 0.001).

S2.4 SoA-Control

Final model

The final mixed model for patients' SoO data retains the fixed effect of *Mode of Movement, Passivity* and *Mode×Passivity* in addition to the random intercept for each subject.

Model diagnostics

Normality of random effects. Shapiro-Wilk test of normality (W = 0.9587, p-value = 0.305).

Normality of conditional raw residuals. Shapiro-Wilk test of normality (W = 0.9868, p-value = 0.031).

S3 Exploratory analyses on antipsychotic dose

S3.1 SoO

Final model

The final mixed model for patients' SoO data retains the fixed effect of *Mode of Movement* and *Olanzapine* in addition to the by-subject random intercept.

Model diagnostics

Normality of random effects. Shapiro-Wilk test of normality (W = 0.9274, p-value = 0.047).

Normality of conditional raw residuals. Shapiro-Wilk test of normality (W = 0.9872, p-value = 0.035).

S3.2 SoA

Final model

The final mixed model for patients' SoA data retains the fixed effect of *Mode of Movement, Olanzapine* and the interaction *Mode of Movement*×*Olanzapine* in addition to the by-subject random intercept.

Model diagnostics

Normality of random effects. Shapiro-Wilk test of normality (W = 0.9786, p-value = 0.802).

Normality of conditional raw residuals. Shapiro-Wilk test of normality (W = 0.9965, p-value = 0.882).

S3.3 SoO-Control

Final model

The final mixed model for patients' SoO-Control data retains only the by-subject random intercept.

Model diagnostics

Normality of random effects. Shapiro-Wilk test of normality (W = 0.9333, p-value = 0.067).

Normality of conditional raw residuals. Shapiro-Wilk test of normality (W = 0.9797, p-value = 0.002).

S3.4 SoA-Control

Final model

The final mixed model for patients' SoA-Control data retains the fixed effect of *Mode of Movement* and the by-subject random intercept.

Model diagnostics

Normality of random effects. Shapiro-Wilk test of normality (W = 0.9709, p-value = 0.586).

Normality of conditional raw residuals. Shapiro-Wilk test of normality (W = 0.9917, p-value = 0.213).

S4 F-test full output, correlations for questionnaire and clinical data, and antipsychotics effect

	df_{num}	df_{den}	F	*p*	
**SoO**					
Mode	1	420	33.309	<.0001	***
Delay	3	420	21.669	<.0001	***
Group	1	59	3.383	0.071	
Delay×Group	3	420	8.091	<.0001	***
**SoA**					
Mode	1	416	243.704	<.0001	***
Delay	3	416	29.124	<.0001	***
Group	1	59	1.038	0.312	
Mode×Delay	3	416	3.961	0.008	**
Mode×Group	1	416	14.807	<.0001	***
Delay×Group	3	416	2.598	0.052	
**SoO-control**					
Mode	1	426	2.898	0.089	
**SoA-control**					
Mode	1	416	176.225	<.0001	***
Delay	3	416	8.613	<.0001	***
Group	1	59	9.572	0.003	**
Mode×Delay	3	416	2.214	0.086	
Mode×Group	1	416	59.706	<.0001	***
Delay×Group	3	416	2.604	0.052	

Supplementary Table 1. Results of Type-III ANOVA on questionnaire scores. * $p < 0.05$, ** $p < 0.01$, *** $p < 0.001$

	df_{num}	df_{den}	F	*p*	
**SoO**					
Mode	1	202	12.819	0.0004	***
**SoA**					
Mode	1	199	165.91	<.0001	***
Delay	3	199	4.684	0.004	**
**SoO-control**					
Mode	1	201	0.031	0.858	
Passivity	1	27	0.561	0.46	
Mode×Passivity	1	201	2.434	0.12	
**SoA-control**					
Mode	1	201	33.497	<.0001	
Passivity	1	27	0.539	0.469	
Mode×Passivity	1	201	6.867	0.009	**

Supplementary Table 2. Results of Type-III ANOVA on questionnaire scores of patients considering passivity symptom severity. * $p < 0.05$, ** $p < 0.01$, *** $p < 0.001$

	df_{num}	df_{den}	F	*p*	
**SoO**					
Mode	1	202	12.819	0.0004	***
Olanzapine	1	27	4.659	0.04	*
**SoA**					
Mode	1	198	166.728	<.0001	***
Delay	3	198	4.707	0.003	**
Olanzapine	1	27	0.49	0.49	
Mode×Olanzapine	1	198	1.981	0.161	
**SoA-control**					
Mode	1	202	32.552	<.0001	***

Supplementary Table 3. Results of Type-III ANOVA on questionnaire scores of patients considering olanzapine equivalent dose. * $p < 0.05$, ** $p < 0.01$, *** $p < 0.001$. Equivalency ratio from Gardner et al. (2010).

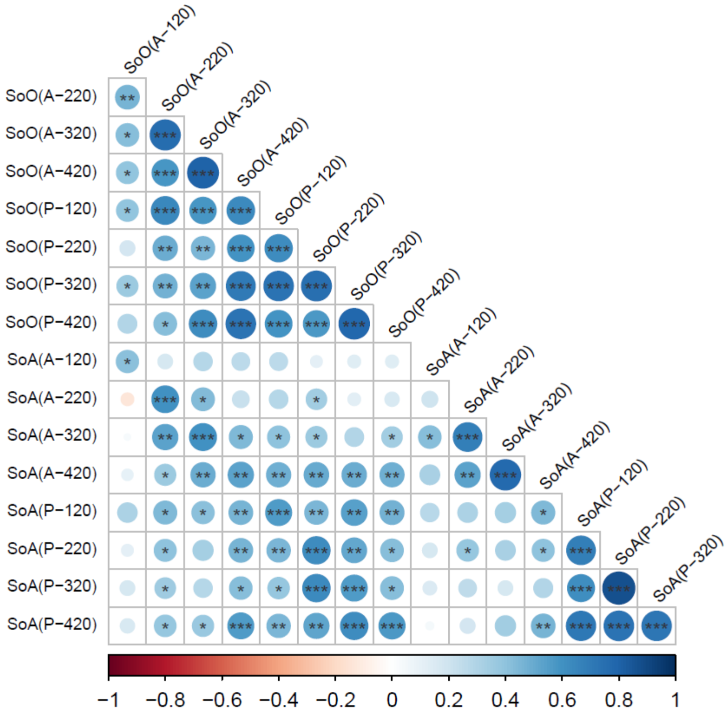

Supplementary Figure 1. Correlation heatmap matrix for SoO and SoA in the control group. A = Active condition, P = Passive condition. Positive correlations are displayed in blue, negative correlations in red. Significance is indicated by the asterisks (*, **, *** for P < .05, P < .01, and P < .001, respectively, uncorrected).

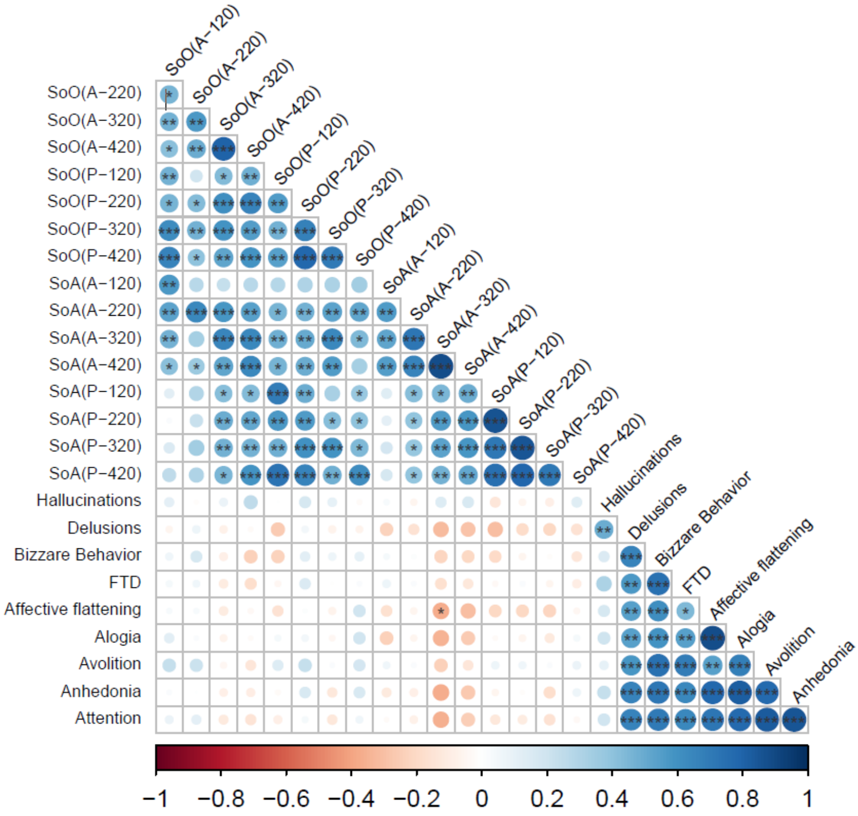

Supplementary Figure 2. Correlation heatmap matrix for SoO, SoA and psychopathological scales in the clinical group. A = Active condition, P = Passive condition. Positive correlations are displayed in blue, negative correlations in red. Significance is indicated by the asterisks (*, **, *** for P < .05, P < .01, and P < .001, respectively, uncorrected).

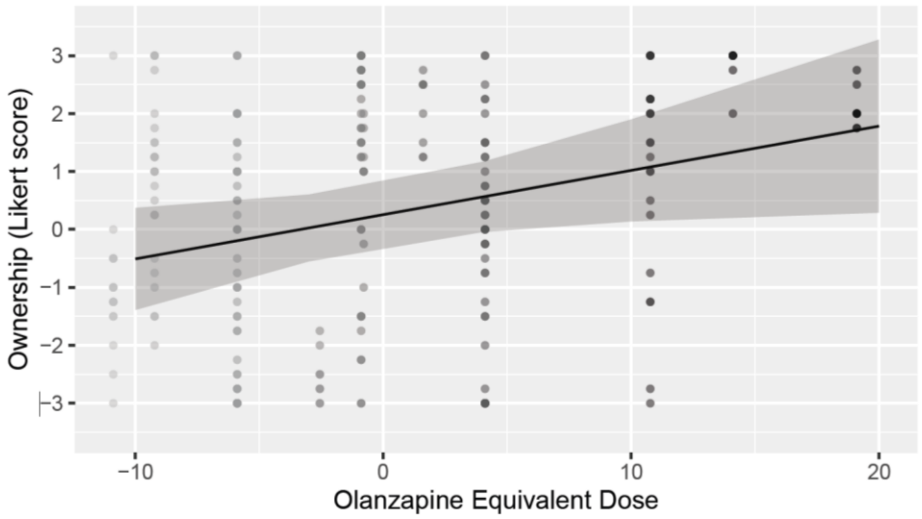

Supplementary Figure 3. Ownership scores: main effect of olanzapine equivalent dose. Lines and point in the foreground represent adjusted means and 95% confidence intervals. Shaded points in the background indicate by-subject ownership mean scores.

S5 Questionnaire

SoO

1. Mi sembrava di guardare direttamente la mia mano oltre lo specchio.
   I felt as if I was looking at my own hand behind the mirror

2. Mi sembrava che la mano nello specchio fosse una parte del mio corpo.
   I felt as if the hand in the mirror was part of my body

3. Mi sembrava che il mio dito reale e il dito nello specchio si muovessero nello stesso posto.
   It seemed as if I my finger and the mirrored finger moved in the same location.

4. Mi sembrava che la mano nello specchio mi appartenesse.
   I felt as if the hand in the mirror was my hand.

SoO-Control

5. Sembrava che la mia mano reale diventasse di gomma.
   I felt as if my real hand were turning rubbery.

6. Mi sembrava di avere più di una mano destra/sinistra.
   It seems as if I had more than one right/left hand.

7. Sembrava che la mano nello specchio si muovesse verso la mia mano.
   It appeared as if the hand in the mirror were drifting towards my real hand.

8. Sembrava che la mia mano destra/sinistra fosse sparita.
   It felt as if my right/left hand had disappeared.

SoA

9. La mano nello specchio si muoveva proprio come volevo, come se stesse obbedendo alla mia volontà.
   The hand in the mirror moved just like I wanted it to, as if it was obeying my will.

10. Sembrava che fossi io a controllare i movimenti della mano nello specchio.
    It seemed as if I was controlling the movements of the hand in the mirror.

11. Sembrava che fossi io a causare i movimenti della mano nello specchio.
    It seemed as if I was causing the movement of the hand in the mirror.

12. Ogni volta che muovevo il mio dito, mi aspettavo di vedere il dito nello specchio muoversi allo stesso modo.
    Whenever I moved my finger I expected the finger in the mirror to move in the same way

SoA-control

13. Mi sembrava che la mano nello specchio stesse controllando la mia volontà.
    It seemed as if the hand in the mirror was controlling my will.

14. Sembrava che la mano nello specchio stesse controllando i miei movimenti.
    It seemed as if the hand in the mirror was controlling my movements.

15. Mi sembrava di sentire i miei movimenti nello spazio tra la mia mano reale e la mano nello specchio.
    I could sense the movement from somewhere between my real hand and the hand in the mirror.

16. Sembrava che la mano nello specchio avesse una sua propria volontà.
    It seemed as if the hand in the mirror had a will of its own.
